# A systems-based method to repurpose marketed therapeutics for antiviral use: a SARS-CoV-2 case study

**DOI:** 10.26508/lsa.202000904

**Published:** 2021-02-16

**Authors:** Mengran Wang, Johanna B Withers, Piero Ricchiuto, Ivan Voitalov, Michael McAnally, Helia N Sanchez, Alif Saleh, Viatcheslav R Akmaev, Susan Dina Ghiassian

**Affiliations:** Scipher Medicine Corporation, Waltham, MA, USA

## Abstract

This study describes two complementary methods that use network-based and sequence similarity tools to identify drug repurposing opportunities predicted to modulate viral proteins. This approach could be rapidly adapted to new and emerging viruses. The first method built and studied a virus–host–physical interaction network; a three-layer multimodal network of drug target proteins, human protein–protein interactions, and viral–host protein–protein interactions. The second method evaluated sequence similarity between viral proteins and other proteins, visualized by constructing a virus–host–similarity interaction network. Methods were validated on the human immunodeficiency virus, hepatitis B, hepatitis C, and human papillomavirus, then deployed on SARS-CoV-2. Comparison of virus–host–physical interaction predictions to known antiviral drugs had AUCs of 0.69, 0.59, 0.78, and 0.67, respectively, reflecting that the scores are predictive of effective drugs. For SARS-CoV-2, 569 candidate drugs were predicted, of which 37 had been included in clinical trials for SARS-CoV-2 (AUC = 0.75, *P*-value 3.21 × 10^−3^). As further validation, top-ranked candidate antiviral drugs were analyzed for binding to protein targets in silico; binding scores generated by BindScope indicated a 70% success rate.

## Abstract (precis)

This study describes complementary network-based and sequence similarity methods to identify drug repurposing opportunities predicted to directly target viral proteins, highlighting results for five human pathogens.

## Introduction

Viruses are obligate intracellular parasites that can only replicate by entering into a host cell and hijacking host cell machinery to produce and assemble new progeny virions. The arsenal to treat viral infections focuses on viral and cellular components that are essential for the viral life cycle. There are two main categories of antiviral therapies: (1) host-targeted antivirals that modulate host proteins that interact with or are influenced by the virus and (2) virus-targeted antivirals that directly bind to and modulate the activity of viral proteins (1). Drugs that target viral proteins may directly limit viral replication and propagation (1).

The medical and scientific communities do not have the luxury of time to develop new compounds that target highly contagious life-threatening viruses with the rapidity required to combat an ongoing pandemic (2). To facilitate the advancement of drugs into clinical trials, identification of drugs that were previously approved for other indications and exhibit a reasonable safety profile represent a resource for potential antiviral therapeutics (3, 4, 5 *Preprint*).

The coronavirus disease 2019 (COVID-19) pandemic, caused by the spread of SARS-CoV-2, has highlighted the need for tools to rapidly identify effective therapies against emerging pathogens. Numerous drugs have been identified that are predicted to alleviate COVID-19 symptoms and are in clinical trials to assess their safety and efficacy (6, 7). Identification of multiple treatment options is important to control the spread of the disease: patients respond differently to the same treatments because of their genetics (8), sequence variations in the viral genome may influence drug efficacy (9), and bottlenecks in drug availability may occur once an effective treatment is identified (10).

One consideration when evaluating drug repurposing opportunities is the ability of a drug to bind to multiple protein targets, called drug promiscuity. It has been previously shown that a drug’s promiscuity is correlated with structural similarity and binding site similarity between the intended and unintended protein targets (11). In this study, network-based tools and bioinformatic approaches identified drug repurposing opportunities that were predicted to directly target viral proteins. The two antiviral drug discovery methods developed in this study were designed to first identify existing protein targets that are structurally similar to viral proteins then, subsequently, predict and rank the interactions likely to occur between existing compounds and viral proteins. These methods were validated using the human immunodeficiency virus 1 (HIV), hepatitis B virus (HBV), hepatitis C virus (HCV), and human papillomavirus (HPV) then used to predict candidate antiviral drug repurposing opportunities for SARS-CoV-2.

## Results

**Figure 1.**
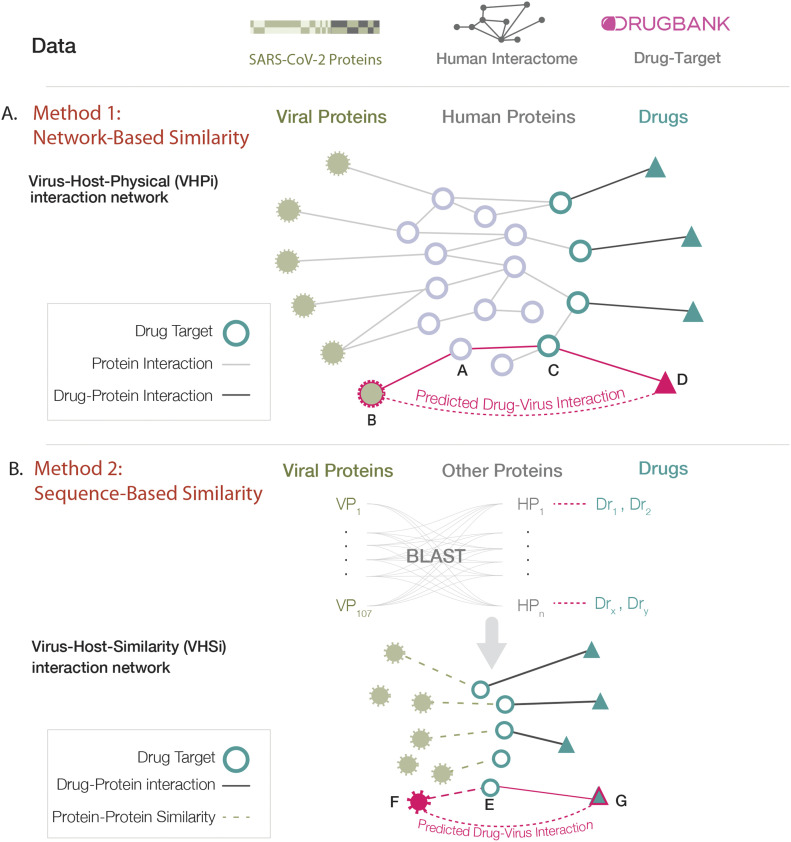
Complementary methods to identify drug repurposing candidates that directly target viral proteins. **(A)** A network-based approach using link prediction to identify drug repurposing opportunities that are at a path length of three from viral proteins on the virus–host–physical interaction network. **(B)** A sequence similarity approach that identifies drug target proteins with protein sequence homology to viral proteins.

In this study, two network- and sequence-based methods were developed to identify candidate drug repurposing opportunities that directly target viral proteins. An underlying assumption behind many drug–target interaction predictions is that structurally similar proteins are more likely to be targeted by similar drugs (11, 12, 13). Using complementary approaches, the methods described herein identify drugs by virtue of homology between viral proteins and proteins that are the known target of therapeutics developed for other indications. Both methods were designed to find proteins that are structurally similar to viral proteins; method 1 inferred structural similarity through common protein–protein interaction (PPI) patterns and method 2 derived structural similarity through sequence homology (Fig 1). When used in combination, these complementary methods derive high-confidence predictions of drug repurposing candidates.

### Systems-based antiviral drug ranking and identification

In the network-based method, the similarity between viral proteins and human proteins was indirectly inferred through their common interaction patterns. We constructed and studied a virus–host–physical interaction (VHPi) network; a three-layer multimodal network of (1) drug and target protein interactions from DrugBank (14,15), (2) consolidated human protein–protein pairwise interactions (Human Interactome, see the Materials and Methods section), and (3) viral–host PPIs (16). In this method, a network-based similarity approach (17) was implemented to find human proteins that are similar to viral proteins. These network-identified proteins were predicted to have similar binding interfaces and thus are likely to interact with the same compounds.

In the VHPi network, identifying a drug that is predicted to bind to a viral protein is a link prediction problem (18) (Fig 1A). In most self-organizing networks, such as social networks, two nodes (people) are more likely to interact with (know) each other if they have a greater number of common neighbors, a phenomenon known as triadic closure principle (19). Hence, most link prediction methods that have been implemented on biological networks use metrics that incorporate common neighbors (20, 21). However, these metrics do not successfully predict interactions in biological networks where interactions occur primarily through physical contacts. For example, protein A interacts with both proteins B and C, independently (Fig 1A). In this case, protein B and C have a shared neighbor–protein A–and are therefore at a network distance of two. The interaction between proteins A and B indicates that these proteins have complementary binding sites, and this assumption holds true for proteins A and C as well. Proteins with a high number of shared neighbors are more likely to have similar binding interfaces and thus tend to interact with the same proteins (nodes). Unlike the case of social networks, proteins B and C are not expected to directly interact with each other. Rather proteins B and C are expected to be structurally similar. Therefore, protein B is predicted to interact with other interacting partners of protein C, in this case protein D. Note that the network distance between protein B and the predicted interacting partner, protein D, is three. Therefore, link prediction applied to biological networks involves finding path lengths of three (L3) (17). Drugs with a path of length three to the viral proteins will have a non-zero rank. In this scoring, drugs with multiple paths of length three to the viral proteins were ranked higher and promiscuous drugs (drugs with a high number of target proteins) tended to have higher scores.

For physical PPI networks, the L3 methodology outperforms other link prediction methods (17). In principle, this can be extended to any network where the interactions are based on physical binding and has the advantage that it should not be limited to one organism or specific node type. In method 1, link prediction can be applied such that drugs known to target human proteins would be expected to interact with viral proteins if the drug is three steps away from a viral protein in the VHPi network (Fig 1A). To generate a ranking score, each drug score was penalized by the degree of the mediator nodes, that is, a protein in the path between a viral protein and predicted drug (see the Materials and Methods section, Equation (1)).

In parallel, a complementary approach was implemented in which the similarity between viral proteins and other proteins was directly derived by calculating their global sequence similarity. In this method, proteins with high sequence similarity were predicted to bind the same small molecules. BLAST homology analysis assessed the amino acid sequence similarity between viral proteins and other protein sequences (see the Materials and Methods section). The results can be visualized by constructing a virus–host–similarity interaction (VHSi) network (Fig 1B).

### Method validation in HIV, HCV, HBV, and HPV

**Figure 2.**
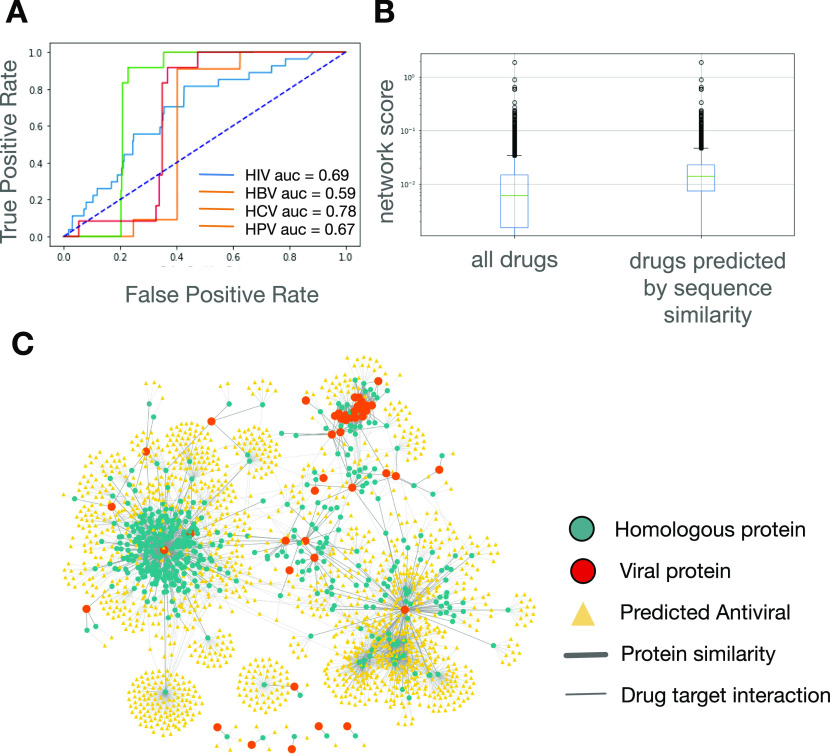
Method validation in HIV, hepatitis B virus, hepatitis C virus, and HPV. **(A)** Receiver operating characteristic curve evaluating predictive power of network-based approach in ranking known drugs in HIV, hepatitis B virus, hepatitis C virus, and HPV. **(B)** Box and whisker plot of network mean scores generated for all drugs with a non-zero value and for drugs identified by the sequence similarity approach for HIV. The sequence similarity approach predicts drugs with high network mean scores. **(C)** Virus–host–similarity interaction network representation of drugs predicted by both methods for HIV.

Four viruses were chosen to validate the methods: HIV, HBV, HCV, and HPV. The VHPi networks were consolidated from (1) drug and target protein interactions from DrugBank (14, 15), (2) consolidated human protein–protein pairwise interactions (Human Interactome, see the Materials and Methods section), and (3) virus–host protein interactions from the National Center for Biotechnology Information (NCBI) HIV-1 Interactions Database (22, 23) and published literature (24, 25, 26) (see the Materials and Methods section; Table S1). Drugs predicted to bind to viral proteins were ranked by calculating the L3 measure of the drug to a viral protein on the VHPi network. From DrugBank, 7,859 drugs (containing approved small molecule drugs, approved biologics, nutraceuticals, and experimental drugs) were considered for which 5,576 (HIV), 4,532 (HBV), 5,313 (HCV), and 5,834 (HPV) drugs were given a non-zero rank based on the L3 measure. To evaluate the performance of ranked drugs, antiviral drugs with at least one human primary target protein were curated from the DrugBank database (See the Materials and Methods section; Table S1). To assess the performance of network, mean scores in predicting antiviral drugs, an ROC curve was generated, which resulted in AUC values of 0.69, 0.59, 0.78, and 0.67 for HIV, HBV, HCV, and HPV, respectively (Fig 2A). This showed that network mean scores were predictive of effective drugs and that other highly ranked drugs represent candidates for further study (Table S2).

Table S1 Virus–host interactions and known antiviral drugs HIV, hepatitis C virus, hepatitis B virus, and HPV.

Table S2 Predicted drugs and associated scores for HIV, hepatitis C virus, hepatitis B virus, and HPV viral proteins.

To reduce the list of predicted drugs, we applied the sequence similarity-based approach described above to the four viruses. BLASTP searches were performed on unique viral protein sequences to find homologous proteins (see the Materials and Methods section). Human proteins homologous to the viral protein sequences were identified for HIV, but not the other viruses. Drugs known to target the homologous proteins were predicted to also bind the HIV viral proteins. Unlike the network-based method where the predictions provide a continuous score for each drug, the predictions made by this complementary method are binary, that is, yes or no. Interestingly, we observed that drugs predicted through sequence-based similarity tended to have high network mean scores as predicted by the network-based method (*P*-value = 3.33 × 10^−22^) (Fig 2B). This observation confirmed the complementary nature of the two approaches. The VHSi network interactions of predicted drugs supported by both methodologies for HIV viral proteins are depicted in Fig 2C. No drug was found through BLAST search for HBV, HCV, and HPV.

### COVID-19: prediction and in silico validation of candidate antiviral drugs

**Figure S1.**
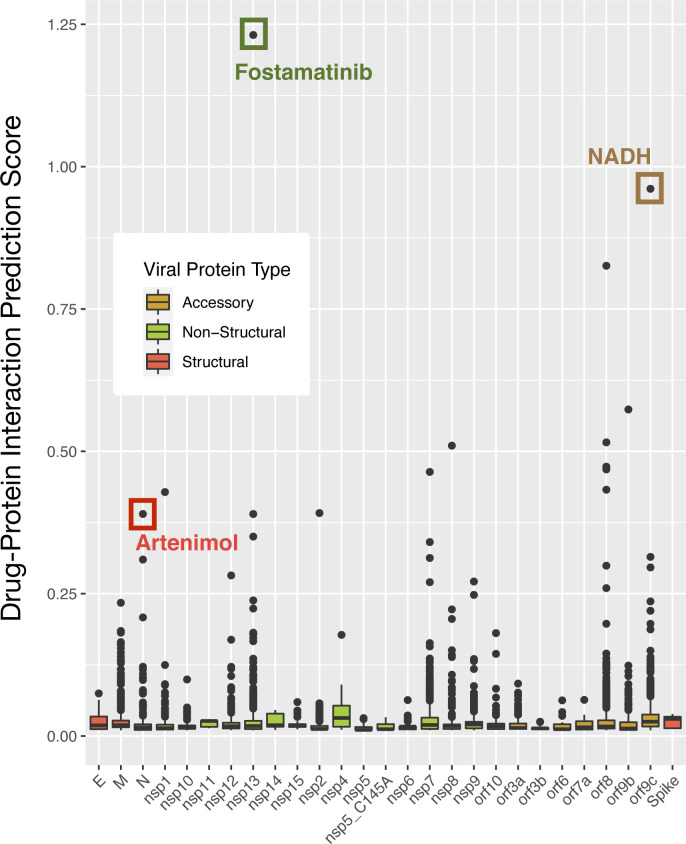
DPI score distribution of predicted antiviral drugs for each SARS-CoV-2 viral protein. Viral proteins are color coded based on the protein class: structural (red), non-structural (green), or accessory (yellow). The drug with the highest score in each viral protein class from method 1 is highlighted.

The SARS-CoV-2 VHPi network was consolidated to identify drugs that are predicted to bind viral proteins based on link prediction. From DrugBank, 7,859 drugs were considered and 4,486 were given a ranking based on the L3 measure of the drug to a viral protein on the VHPi network. The highest-ranked drug for each viral protein type (structural, non-structural, and accessory) is shown in Fig S1. The antimalarial drug artenimol was predicted to target the nucleocapsid structural protein, the tyrosine kinase inhibitor fostamatinib used to treat chronic immune thrombocytopenia was predicted to target the non-structural protein Nsp13, and the coenzyme NADH was predicted to bind to the viral protein ORF9C.

**Figure 3.**
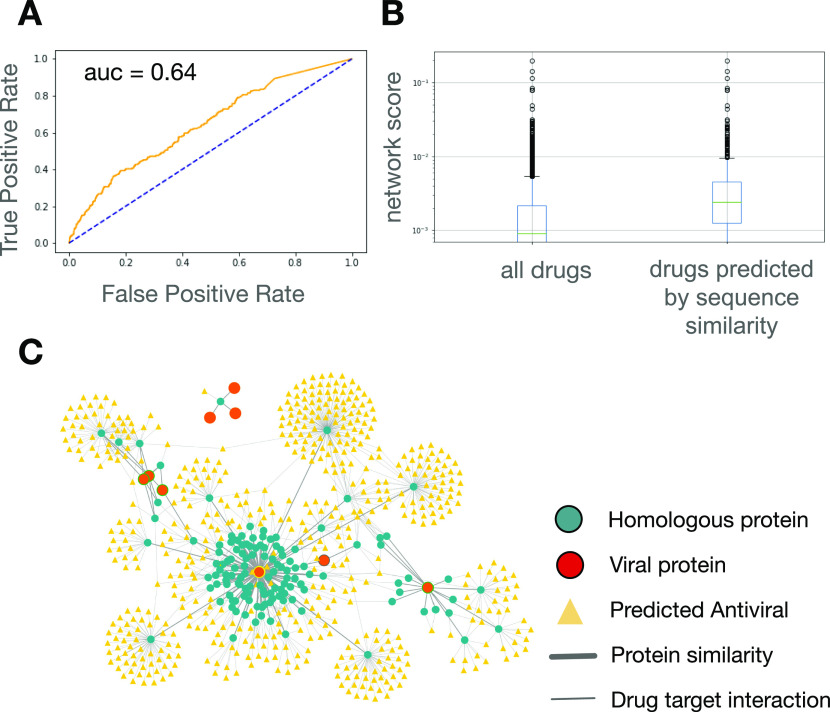
Method implementation on SARS-CoV-2. **(A)** Receiver operating characteristic curve evaluating predictive power of network-based approach in ranking drugs currently under investigation for COVID-19 in clinical trials. **(B)** Box and whisker plot of network mean scores generated for all drugs with a non-zero value and for drugs identified by the sequence similarity approach. **(C)** Visualization of predicted antiviral drugs and SARS-CoV-2 Protein Data Bank structures on the virus–host–similarity interaction network.

As there are currently no approved drugs for COVID-19, we used a list of drugs currently in clinical trials for preliminary validation (Table S3). A list of 322 drugs in clinical trials for COVID-19 (as of 1 January 2021) was curated from ClinicalTrials.gov as a surrogate for effective drugs (see the Materials and Methods section). The drug rankings provided by the network-based approach were able to predict the drugs in clinical trials with an AUC of 0.64 (Fig 3A).

Table S3 SARS-CoV-2 virus–host interactions and drugs currently in clinical trials for COVID-19.

To refine the drug predictions, sequence-based similarity analyses of SARS-CoV-2 viral proteins were performed. To facilitate further in silico methodology validation, the similarity analyses were restricted to those proteins for which a structure was available in the Protein Data Bank (PDB). Similar to HIV, the drugs predicted by sequence similarity tended to have high scores as predicted by the network-based method (*P*-value = 2.57 × 10^−38^) (Fig 3B). The interaction patterns of the human proteins with sequence homology to SARS-CoV-2 viral proteins were visualized on the VHSi network (Fig 3C). The network visualization represents 569 SARS-CoV-2 candidate antiviral drugs predicted by the sequence similarity method that had a non-zero network mean score according to the network-based method (Table S4). Among these 569 predicted drugs, 37 of them have been included in clinical trials for SARS-CoV-2 (AUC = 0.75, *P*-value 3.21 × 10^−3^). Gene ontology analysis of human proteins that are homologous to SARS-CoV-2 proteins indicated an enrichment for proteins involved in neurotransmission and mitochondrial function (Table S5).

Table S4 Predicted drugs and associated scores for SARS-CoV-2 viral proteins.

Table S5 Gene ontology terms associated with human proteins homologous to SARS-CoV-2 viral proteins.

Computational prediction of drug–target interactions has become an essential step in the drug discovery process. The BindScope web application for active-inactive classification of compounds based on deep convolutional neural networks (27), was used to evaluate the binding affinity of the candidate drug–protein pairs predicted in this study. The interactions are scored based on probability where values close to one imply strong binding affinity and those close to zero imply low binding affinity. Binding scores were determined for the drug–targets pairs predicted by both the network-based and sequence similarity methods introduced in this study (Table 1). The results showed that 70% of the predicted drug-virus pairs have a binding score over 0.6.

**Table 1.** Binding scores for drug–targets pairs predicted by network-based and sequence similarity methods.

Drug name	Drugbank ID	Viral protein name	Viral protein target PDB	Network mean score	BindScope score
Artenimol	DB11638	NSP8	7BTF_chainB	0.51001897	0.951099694
NADH	DB00157	NSP13	7BTF_chainC	0.35027915	0.908634782
Fostamatinib	DB12010	NSP13	7BTF_chainC	1.23132454	0.878183186
Artenimol	DB11638	N	6YI3	0.38998956	0.793080389
Fostamatinib	DB12010	ORF9b	6Z4U	0.57350959	0.535310388
NADH	DB00157	ORF8	QHD43422	0.43256958	0.407533526
Fostamatinib	DB12010	NSP7	6WCF	0.31270554	0.27964434
Fostamatinib	DB12010	NSP1	QHD43415_1	0.42845161	0.1326
NADH	DB00157	NSP7	6WCF	0.46385305	0.034212183
Fostamatinib	DB12010	NSP2	QHD43415_2	0.39158755	0.0123
Fostamatinib	DB12010	ORF8	QHD43422	0.4730801	0.01216421
Fostamatinib	DB120109	N	6YI3	0.30990618	0.003463759

Colors indicate strength of binding predictions from BindScope from poor (red) to good (green).

## Discussion

Using network topological and biological properties of viral proteins, two complementary methods were developed for identification of candidate antiviral therapies. Host proteins that interact with viral proteins were studied by mapping the proteins onto a consolidated Human Interactome map of PPIs, observing their underlying interaction patterns, and studying their biological properties. Four well-studied viruses were used to validate the drug prediction methods. Candidate antivirals ranked by the network-based method predicted known HIV, HBC, HCV, and HPV drugs with AUC values of 0.69, 0.59, 0.78, and 0.67, respectively, reflecting that the network mean scores were predictive of effective drugs. Finally, the novel SARS-CoV-2 drug–virus protein interactions that were predicted by both methods were validated in silico using BindScope and resulted in a 70% success rate of identifying candidate antiviral therapies that bind directly to viral proteins with high affinity. This suggests that the methods described herein have the potential to identify candidate drug repurposing opportunities that directly bind to viral proteins.

Numerous antiviral drug repurposing candidates identified by this study were predicted to bind to viral proteins using BindScope. The application of deep neural networks through tools such as BindScope has opened a new path to perform molecular docking using methods that have been previously trained and validated to predict drug–target interactions. Many of the candidate antiviral drugs were predicted to target SARS-CoV-2, which reflects the ability of the methods to identify promiscuous drugs. However, inclusion of the sequence similarity-based method eliminated many drugs that bind indiscriminately and lack specificity for the particular virus of interest.

Gene ontology analysis of the proteins homologous to SARS-CoV-2 proteins identified enrichment of terms associated with mitochondria and synaptic transmission. Previous studies suggested a link between SARS-CoV-2 pathogenesis and manipulation of mitochondrial function (16, 28, 29). Homology to human mitochondrial proteins may enable viral proteins to bind to and manipulate mitochondrial protein function to promote viral entry and modulate host responses to viral infection (29). The enrichment of gene ontology terms associated with neurological processes in consistent with the neuroinvasive potential of SARS-CoV-2 (30, 31, 32). Patients with COVID-19 show signs of neurologic involvement including headache, loss of taste and smell, or, less frequently, encephalopathy and acute cerebrovascular disease (33, 34). The homology between human proteins and those encoded by SARS-CoV-2, suggests that, as is common among viruses, mimicry of host proteins can promote infection and pathogenesis. Although the similarity between viral and host proteins can make it difficult for the immune system to recognize and clear invading pathogens, this same characteristic could be exploited to identify drug repurposing opportunities.

Among the top predicted HIV-1 drug/target combinations are drugs approved by the FDA for treatment of HIV (etravirine, zidovudine, and lamivudine) and have completed Phase III clinical trials (bictegravir) (35). A fifth drug, capravirine, was predicted in this study to target HIV-1 Gag protein; however, its development ceased because of safety concerns and because it did not provide sufficiently substantial improvement over already available treatment options. Among the top predicted SARS-CoV-2–specific drug repurposing candidates were fostamatinib, glutathione, and valproic acid. Fostamatinib, artenimol, and bosutinib were identified in other bioinformatic studies as a potential treatment option to target human proteins associated with COVID-19 (36, 37). Furthermore, fostamatinib is used to treat chronic immune thrombocytopenia, a condition associated with severe COVID-19 (38). Glutathione is an antioxidant that helps defend against oxidative damage of cells from reactive oxygen species and regulates metabolic pathways vital to whole-body homeostasis (39, 40). Glutathione deficiency in patients has been linked to severe manifestations of COVID-19, which has been attributed to indirect effects of glutathione deficiency on enhancing SARS-CoV-2–induced lung damage (41). The results of this study suggest that glutathione may also interact with viral proteins. Valproic acid is a short chain fatty acid with antiviral activity against several viruses (42, 43) and might also be effective against SARS-CoV-2 (44, 45, 46). Therefore, the network- and sequence similarity-based methods identified compounds that may limit viral activities.

Viral proteins often share common tertiary folds and short segments of protein sequence homology with proteins encoded by the host that they infect. These similarities can be exploited to identify drugs that may limit viral replication. However, subtle differences in amino acid sequences relative to the human protein or mutation of viral genomes can limit the efficacy and affinity of a drug for a viral protein by altering binding affinity or binding site availability. World-wide mutations of SARS-CoV-2 have been studied extensively in other publications, and the mutation rate of the virus is considered to be low (47, 48, 49, 50). Mutations that result in amino acid changes are the most common mutation type and every viral protein has at least one frequently recurring mutation (48). Preclinical testing of any candidate viral protein drug target should include assessments of these common protein sequence variations. The SARS-CoV-2 protein with the top-ranked drug repurposing opportunities was Nsp15. Nsp15 is of particular interest as a drug target because it is an endoribonuclease common to all coronaviruses, is necessary for SARS-CoV-2 replication and interferes with host immune responses (51, 52, 53, 54, 55, 56, 57).

Many of the predicted drug repurposing candidates identified by these methods were molecules that promiscuously bind to many proteins and are required for a sizable fraction of all human enzymatic activities (e.g., NADH, zinc, pyridoxal phosphate, and serine) (58, 59, 60, 61). The ability to identify promiscuous drugs is both the greatest strength and greatest weakness of the methods described herein. Therefore, experimental evidence is required to assess whether any of the candidate antiviral drugs reported in this study possess the ability to affect the SARS-CoV-2 replication cycle. This work does not directly test the efficacy of these drugs in experimental models or in clinical trials. It does, however, identify drugs that represent promising repurposing candidates for further study.

## Materials and Methods

### Human interactome

The Human Interactome was consolidated as previously described (62, 63) from 21 public databases containing different types of experimentally derived PPIs data:

1. Binary PPIs, derived from high-throughput yeast-two hybrid (Y2H) experiments (HI-Union [2016] (64)), 3D protein structures (Interactome3D (65), Instruct (66), Insider (67)) or literature curation (PINA [2014] (68), MINT [2019] (69), LitBM17 [2013] (64), Interactome3D, Instruct, Insider, BioGrid [2019] (70), HINT [2019] (71), HIPPIE (72), APID (73), InWeb (74), IntAct (75))
2. PPIs identified by affinity purification followed by mass spectrometry present in BioPlex2 (2017) (76), QUBIC (77), CoFrac (78), HINT, HIPPIE, APID, LitBM17, and InWeb
3. Kinase-substrate interactions from KinomeNetworkX (79) and PhosphoSitePlus (80)
4. Signaling interactions from SignaLink (2019) (81) and InnateDB (2019) (82); and
5. Regulatory interactions derived by the ENCODE consortium (2012).

The curated list of molecular interactions provided by Alonso-López et al (73) was used for differentiating binary interactions among the experimental methods present in the literature curation databases. For InWeb, interactions with a curation score <0.175 (75^th^ percentile) were not considered. All proteins were mapped to their corresponding Entrez ID (NCBI), with unmapped proteins removed. The resulting interactome includes 18,505 proteins and 327,924 interactions.

The network analyses were limited to the largest connected component, containing 18,446 proteins and 322,159 interactions.

### Viral–host interactions curation and analysis

The map of virus–host interactions for SARS-CoV-2 was generated using an affinity purification mass spectrometry experiment (16). Virus–host interactions with high-confidence scores were selected, resulting in 332 interactions among 26 viral proteins and 332 unique human proteins (Table S3).

The entire list of submitted HIV-1 virus–host interactions was downloaded from NCBI website (Table S1).

As the network-based link prediction method was based on physical interactions of the nodes (proteins), interactions were limited to physical interaction annotations only. The interaction types considered as physical included:

1. Physical binding interactions: interacts with, associates with, complexes with, fractionates with, interacts, binds, and
2. Substrate–enzyme interactions: deglycosylated, glycosylated by, modified by, processed by, sulfated by, methylated by, palmitoylated by, is polyubiquitinated by, phosphorylated by, recruited by, ubiquitinated by, isomerized by, myristoylated by, acetylated by, sumoylated by, phosphorylates, acetylates, dephosphorylated by, and ubiquitinates.

Virus–host interactions included in analyses had at least two sources of evidence (https://www.ncbi.nlm.nih.gov/genome/viruses/retroviruses/hiv-1/interactions/). Virus–host interactions for HBV, HCV, and HPV were as reported in the literature (24, 25, 26) (Table S1).

### Method 1: link prediction method (L3)

The link prediction problem between two nodes describes the existence of network paths of length three (L3) between the two nodes. To account for biases resulting from high-degree nodes (i.e., those with a large number of connections on the Human Interactome), a degree-normalized version of L3 was used where the score of each drug–target pair was penalized by the degree of the nodes in the path of length three between them:

$$P_{d,t}=\sum_{i,j}\frac{a_{d,i}a_{i,j}a_{j,t}}{\sqrt{k_{i}}k_{j}}$$ (1)

Where *k*_*i*_ represents the degree of node i and *a*_*i,j*_ = 1 if nodes i and j interact, and zero otherwise.

### Drug protein interaction (DPI) data sources

The protein targets for the drugs evaluated in this study were obtained from DrugBank Release Version 5.1.6 (https://github.com/emreg00/drugbox) (14, 15). Only drugs with at least one reported target were included in this analysis. For network-based rankings, drugs were required to have at least one protein target in the human interactome (6,128 drugs in total).

### Curation of COVID-19 drugs in clinical trial and known HIV drugs

Using the ClinicalTrials.gov Advanced Search tool, all studies with the condition or disease corresponding to either COVID-19, SARS-CoV-2, or 2019-nCoV were retrieved. Condition terms were those designated by ClinicalTrials.gov to include all COVID-19 studies. Similar to the clinical trials curation procedure from a previous study (5 *Preprint*), we selected clinical studies of interventional type, and whose fist submission year was not earlier than 2020. Studies that are either terminated, suspended, no longer available or withdrawn were excluded from the analysis. For each remaining study, drug names were retrieved by parsing the “intervention_name” and “mesh_term” fields corresponding to interventions used in a study. To search these fields, we manually constructed a list of regular expressions containing synonyms, possible misspellings or non-English names of drugs. Drugs presented in the “intervention_name” and “mesh_term” fields were then checked against the compiled list of regular expressions, and each study was mapped to a list of drugs used in it. Finally, we compiled a list of potential COVID-19 drugs that were used in clinical trials at least once for all studies submitted as of 1 January 2021.

The list of known drugs for HIV were selected manually from the literature and the corresponding targets were curated from DrugBank. A list of 54 “known HIV drugs” was generated from approved HIV drugs and drugs currently in late stage clinical trials (Phase II and Phase III) curated from the DrugBank database on 22 May 2020. A distinction was made between drugs with primary targets (polypeptides), which are thought to modulate the therapeutically beneficial effects of each drug, and drugs with secondary targets (enzymes, transporters, and carriers), which mediate pharmacologically relevant effects (83). Of the 54 curated known HIV drugs, 27 had at least one human primary target. Drugs targeting HBV, HCV, and HPV were obtained from DrugBank.

### Method 2: curation of protein FASTA sequences from PDB and BLAST sequence similarity analysis

160 SARS-CoV-2 protein structures and corresponding amino acid sequences were obtained from the PDB. Redundant amino acid sequences were removed, resulting in 149 unique sequences.

HIV-1, HBV, HCV, and HPV protein structures and corresponding amino acid sequences were obtained from the PDB. After filtering the “Entity Macromolecule Type” to “Polypeptide only” to remove nucleic acid sequences, a list of 3,421 amino acid sequences was generated for HIV-1. A list of 30, 238, 47 amino acid sequences was generated for HBV, HCV, and HPV, respectively. BLAST search found only a few homologous proteins and none of them are known drug targets.

Because of the large number of existing HIV amino acid sequences, redundant sequences, as measured by pairwise sequence similarities, were removed. The 3,421 sequences were split into four batches, each with a similar number of sequences, to generate a pairwise distance matrix for each batch using the MSA package in R (84). An empirical cutoff of 0.8 was selected from the distribution plot and used to remove one of the two paired sequences with distance below 0.8 (high similarity). After processing all four batches, the total number of amino acid sequences was reduced to 363. MSA was run again on the reduced list of 363 sequences from all four batches. Using the same distance cutoff of 0.8, the final number of unique sequences was reduced to 199.

The protein FASTA sequences were used as input for the similarity searching program BLASTP from NCBI with standard general parameters and “max target sequences” set to 20,000 non-redundant (nr) protein sequences. Because finding distantly related protein sequences is more challenging than finding closely related sequences, the BLOSUM62 matrix (85) was used. NCBI provides a unique accession ID for each homolog protein found in the BLAST search results. NCBI accession IDs were converted to a UniProt ID using the ID Mapping database (https://ftp.expasy.org/databases/uniprot/current_release/knowledgebase/idmapping/).

BLASTP was performed with the final list of unique viral protein sequences querying human (specifying homo sapiens in “Organism”) and non-human (excluding homo sapiens in “Organism”) sequences to find proteins with similarity to the viral proteins (see Supplemental Data 1).

Supplemental Data 1. Virus-host interactions and known antiviral drugs HIV, HCV, HBV and HPV

### Evaluating performance of drug predictions

Each drug was assigned by method 1 (network-based similarity method, Fig 1) a score specific to each viral protein. The drug’s mean score across all viral proteins was used as the final score and method validation. Performance of the drug predictions was evaluated by area under the receiver operating characteristic curve (AUC), which was calculated by comparing network mean scores provided by network-based method 1 and the label indicating whether the predicted drug was used in a clinical trial for COVID-19 or a known antiviral drug with at least one human primary target (see Supplemental Data 1).

### DPI scores

BindScope predicts DPIs for a given protein PDB structure and small molecule (e.g., drug) (27). The binding pocket and ligand pose are featurized by voxelizing according to different pharmacophoric-like properties, then trained on a three-dimensional convolutional neural network to predict the binding likelihood. BindScope was trained on ligand conformations from the latest iteration of the Directory of Useful Decoys, Enhanced (DUD-E) (86), and docked using Smina (87). In their article, the authors evaluated BindScope performance using AUC and per receptor fivefold cross-validation. The yielded AUC values ranged from 0.496 to 0.997 with 0.885 as the average.

To use BindScope, PDB structures were downloaded from the PDB database, water molecules were removed and one polypeptide chain was extracted for the analysis. The protein hydrogens were added to the PDB structure in BindScope. Ligand structures were downloaded from DrugBank in SDF format.

## Data Availability

https://github.com/ScipherMedicine/NetworkMedicine.

## Supplementary Material

Reviewer comments
