# Supplementary material for: A systems-based method to repurpose marketed therapeutics for antiviral use: a SARS-CoV-2 case study
Source: Life Sci Alliance. 2021 Feb 16;4(5):e202000904. doi: 10.26508/lsa.202000904 (PMC7893815; doi:10.26508/lsa.202000904)
Supplement: Supplementary file 6 [file LSA_LSA-2020-00904_Supplemental_Data_1.docx]

SUPPLEMENTARY METHODS

Human and Non-Human Search

Starting with a list of HIV-related viral proteins from PDB database, human (by only including “homo sapiens” as organism) and non-human (by excluding “homo sapiens” in organism) searches were performed in NCBI BLASTP to get all amino acid sequences which are similar to the HIV related viral proteins. The results from the two searches were combined and filtered by including only human proteins as the final result (specifying NCBI taxonomy id for homo sapiens:9606). Filtering was necessary as most of the non-human proteins in the BLASTP results were HIV-related proteins; this would have led identifying drugs already targeting HIV, rendering the validation invalid.

For HIV, 1,595 drugs were found using the human search parameters, 1,557 of them have at least one human target, whereas in the non-human search, 949 drugs were found. One drug was removed for having no human protein targets. This indicates that the human-only search may be sufficient to find drugs with at least one human protein target for validation purposes.

For SARS-CoV-2, 621 drugs were found by the human-only search and 12 drugs by the non-human search. All 621 drugs found by the human search had at least one human target, whereas none of the 12 drugs found by the non-human search had human targets. The 30 drugs used in clinical trials for COVID-19 were only found by the human search. Therefore, the non-human search introduced 12 novel candidate drugs.

HIV Performance Evaluation

By definition, the network-based method was only able to predict drugs with at least one human target. Since this method used the similarity of the interaction patterns between two proteins, it was essential to include reliable physical drug-target interactions. Therefore, for validation, the search area was limited to those drugs with at least one primary human target.

To evaluate the performance of method 1 results, the results with approved HIV drugs was compared to drugs with human protein targets. The protein target information for 54 approved HIV drugs was retrieved from DrugBank. A list of 27 drugs with at least one human protein as the primary target (human polypeptides) was generated. The AUC was calculated by comparing network mean scores from method 1 and the label indicating whether they were among the 27 approved drugs with at least one human primary target.

Accessing Drugs Used in Clinical Trials from the [ClinicalTrials.gov](http://ClinicalTrials.gov) Website

Studies with condition or disease corresponding to either COVID-19 OR SARS-CoV-2 OR 2019-nCoV were requested from the [ClinicalTrials.gov](http://ClinicalTrials.gov) Advanced Search tool. Condition terms were those designated by [ClinicalTrials.gov](http://ClinicalTrials.gov) to include all COVID-19 studies. The “intervention_name” and “mesh_term” fields in the returned observational studies submitted not earlier than 2020 were then mined in Python with regular expressions. The “intervention_name” and “mesh_term” fields contain a description of the interventions for the study-including drugs if applicable. Each study was assigned a list of drugs used in it upon checking the intervention fields against the manually curated list of regular expressions containing synonyms, misspellings, and non-English names corresponding to the drug.

An example of regular expression used to identify if a drug was used in a study:

re.search(“hydroxychloroquine|plaquenil|^hcq|hidroxicloroquina|hydoxychloroquine|hydroxycloroquine,” intervention_string.lower())

This string search returns True if an “intervention_string” retrieved either from the “intervention_name” or “mesh_term” fields matches regular expression patterns corresponding to Hydroxychloroquine. The code example uses the “re” module provided as a part of standard Python 3 library.

We compiled a list of potential COVID-19 drugs that were used in clinical trials at least once for all studies submitted as of 1 Jan 2021.
